# Extended opening hours and patient experience of general practice in England: multilevel regression analysis of a national patient survey

**DOI:** 10.1136/bmjqs-2016-005233

**Published:** 2016-06-24

**Authors:** Thomas E Cowling, Matthew Harris, Azeem Majeed

**Affiliations:** 1 Department of Primary Care and Public Health, Imperial College London, London, UK; 2 Centre for Health Policy, Imperial College London, London, UK

**Keywords:** Primary care, Patient satisfaction, Health policy, Health services research, General practice

## Abstract

**Background:**

The UK government plans to extend the opening hours of general practices in England. The ‘extended hours access scheme’ pays practices for providing appointments outside core times (08:00 to 18.30, Monday to Friday) for at least 30 min per 1000 registered patients each week.

**Objective:**

To determine the association between extended hours access scheme participation and patient experience.

**Methods:**

Retrospective analysis of a national cross-sectional survey completed by questionnaire (General Practice Patient Survey 2013–2014); 903 357 survey respondents aged ≥18 years old and registered to 8005 general practices formed the study population. Outcome measures were satisfaction with opening hours, experience of making an appointment and overall experience (on five-level interval scales from 0 to 100). Mean differences between scheme participation groups were estimated using multilevel random-effects regression, propensity score matching and instrumental variable analysis.

**Results:**

Most patients were very (37.2%) or fairly satisfied (42.7%) with the opening hours of their general practices; results were similar for experience of making an appointment and overall experience. Most general practices participated in the extended hours access scheme (73.9%). Mean differences in outcome measures between scheme participants and non-participants were positive but small across estimation methods (mean differences ≤1.79). For example, scheme participation was associated with a 1.25 (95% CI 0.96 to 1.55) increase in satisfaction with opening hours using multilevel regression; this association was slightly greater when patients could not take time off work to see a general practitioner (2.08, 95% CI 1.53 to 2.63).

**Conclusions:**

Participation in the extended hours access scheme has a limited association with three patient experience measures. This questions expected impacts of current plans to extend opening hours on patient experience.

## Introduction

‘The public now expect a seven day NHS’, asserted the UK health secretary in parliament on 15 September 2015.^1^ He was explaining government policy for all general practices in England's National Health Service (NHS) to offer routine doctor appointments 7 days a week, from 08:00 to 20:00, by 2020 (table 1a).^2^ The prime minister first announced this commitment ahead of the 2015 UK general election and restated it in his first major speech after being re-elected.^3^ ^4^ Policy rhetoric focuses on ‘hard working taxpayers and families’ and ‘appointments that fit in with their family and work life’.^5–7^ The government planned for 18 million patients (33% of the population) from 2500 general practices to benefit by March 2016, with national implementation to follow.^8^ This is part of a wider drive to improve weekend services across the NHS, including in hospitals, and is particularly relevant to people who cannot take time off work.^4^ New appointments can be given by telephone or online and provided collectively between practices working in groups.^8^ Most practices currently offer appointments between 08:00 and 18:30, Monday to Friday only.^9^ The Royal College of General Practitioners opposes the plans (table 2).^10^

**Table 1:** National policies to extend the opening hours of general practices in England

	(a) Prime minister's GP access fund	(b) Extended hours access scheme
What is the policy?	Dedicated funding of local schemes designed to improve access to general practice. Practices must offer routine appointments from 08:00 to 20:00 on weekdays and improved access at weekends. Some schemes involve practices working in groups to provide additional appointments and the use of telephone and online consultations. The prime minister has pledged that all general practices will offer routine appointments from 08:00 to 20:00, 7 days a week, by 2020.	Payment for general practices providing appointments outside of times included in contracts (08:00 to 18:30, Monday to Friday for most practices). Practices must provide at least 30 min of additional appointments per 1000 registered patients weekly. Appointments can be with any health professional and must be in addition to normal provision during contracted hours. Sessions can be provided concurrently, for at least 30 min. Participating practices earn £1.90 per registered patient per year.
When was it introduced?	First wave of pilots planned from October 2013 to April 2014; second wave planned from September 2014 to March 2015.	2008; revised for 2014–2015 (to allow practices to offer telephone and online appointments and work in groups to meet requirements).
How many practices have participated?	Around 2517 (1100 from first wave; 1417 from second wave).	5877 (of 7959; 74%) in 2014–2015.
What has been the effect?	Mixed evidence from relevant evaluations in Greater Manchester and London; low demand in some areas.	Unknown.
How much does it cost?	£175 million investment so far (£50 million first wave; £125 million second wave).	£84 million per year (2014–2015 figure).

General practices in England have registered populations of patients for whom they are contracted to provide primary care. They provide comprehensive, continuous services and are generally the first point of contact within the system. Most practices are contracted to open from 08:00 to 18.30, Monday to Friday. Outside of these times, separate out of hours services are available; these vary widely but often include telephone-based care. Urgent and emergency care services range from consultant-led emergency departments to general practitioner or nurse-led services intended to treat minor illnesses that are accessible without appointment. Other services include a national telephone helpline and pharmacists.

**Table 2:** Department of Health's rationale for 7-day general practice services and the concerns of the Royal College of General Practitioners

Department of Health^1^ ^2^ ^7^	Royal College of General Practitioners^10^
▸ This is about responding to the fact that the public now do expect a seven day NHS ▸ The role and purpose of seven day primary care is about much more than convenience—it is about making sure precious hospital capacity is kept clear for those who really need it ▸ This is a manifesto commitment that this government made, so we have to honour that, but it's part of a much bigger strategy which is a massive increase in the capacity of general practice ▸ We live in a 24/7 society, and we need GPs to find new ways of working so they can offer appointments at times that suit hard-working people	▸ Evidence that seven day access is being called for by patients, or that it provides an effective use of NHS resources is, at best, mixed ▸ It is unrealistic to talk about extending routine services at the current time because general practice is hugely overstretched and under-resourced ▸ The promise of seven day access to routine GP care has further damaged morale and is likely to discourage many medical graduates from choosing general practice ▸ We are concerned that the proposal to provide seven day GP access to routine care could jeopardise continuity of care

Text is directly quoted from the given references.

GP, general practitioner; NHS, National Health Service.

Other countries are also trying to improve access to primary care outside of current working hours. Australia recently reintroduced a national funding scheme for after-hours care where practices are paid more for directly providing services outside of 08:00 to 18:00 on weekdays.^11^ An Italian law passed in 2013 intended for practices to work in groups to provide care 24 hours a day, 7 days a week, as part of a wide reorganisation of primary care.^12^ Standards for the ‘patient-centred medical home’ promoted as the basis for primary care reform in the USA include extended opening hours in the evenings and at weekends.^13^

General practices in England have been paid for extended opening hours under a dedicated scheme since 2008 (table 1b). The ‘extended hours access scheme’ pays practices for providing at least 30 min of additional appointments per 1000 registered patients each week outside of the times specified in their main contracts.^14^ Practices earn £1.90 ($2.89; €2.69) per registered patient per year for meeting this requirement.^15^ A practice with the mean number of registered patients (7426^16^) receives £14 109 for providing at least 3 hours and 45 min of extended opening hours per week. The total payment nationally was £84 million in 2014–2015, with 74% (5877/7959) of practices participating.^17^ This investment is similar to that outlined for 7-day opening of general practices, for which a £400 million commitment over the five years to 2020 has been made (table 1).^3^

Practices participating in the scheme are advised to set their opening hours using results from the General Practice Patient Survey—an annual national study of adults registered with a general practitioner (GP).^18^ ^19^ Overall experience and experience of making appointments as reported in the survey are monitored nationally as part of the NHS outcomes framework.^20^ Questionnaires also ask about patient satisfaction with opening hours. This provides the opportunity to examine the association of the extended hours access scheme with several patient experience measures that national policies presume will be affected by extended opening hours, particularly for people unable to take time off work. No studies have previously determined this association. The impact of opening 7 days a week is also largely unknown.

We examined whether patients registered to general practices participating in the extended hours access scheme report a better patient experience across three measures from the General Practice Patient Survey—satisfaction with opening hours, experience of making an appointment and overall experience. We also examined whether the associations varied by patient ability to take time off work to see a GP.

## Methods

### Patient experience

The General Practice Patient Survey 2013–2014 (July to September 2013 and January to March 2014) included all general practices in England with eligible patients (n=8017).^21^ Adults with a valid NHS number and registered to a general practice for at least six months were eligible to participate in the survey. Postal questionnaires were sent to stratified (by age group, gender and practice) random samples of eligible patients in each practice, with 903 357 responses for 8005 practices (34.3% of 2 631 209 questionnaires sent). The mean number of 113 responses per practice (SD 18.5) provides most measures of patient experience with practice-level reliability that is ‘very good’ (≥0.85) or ‘excellent’ (≥0.90).^22^ The weighted respondent sample, accounting for survey design and non-response (by variables including age, gender, socio-economic status, general practice and region of England), is nationally representative.^21^

We analysed three patient experience domains—satisfaction with opening hours, experience of making an appointment and overall experience. Each domain was assessed using a single survey question with five response options. Satisfaction with opening hours was recorded as very dissatisfied, fairly dissatisfied, neither satisfied nor dissatisfied, fairly satisfied or very satisfied. Experience of making an appointment and overall experience were recorded as very poor, fairly poor, neither good nor poor, fairly good or very good. We treated these responses, like in previous research, as lying on an interval scale: 0 (least favourable), 25, 50, 75, 100 (most favourable).^23–25^ All respondents were asked to complete the questions analysed. The three domains address opening hours specifically and patient experience as monitored by UK government.^20^

### Extended opening hours

Payments made to each general practice under the extended hours access scheme 2013–2014 were obtained from the Health and Social Care Information Centre.^26^ Practice-level data on general practice payments became available for the first time in February 2015 and are not provided for financial years (April to March) before 2013–2014. Payments are extracted from general practice computer systems and validated against statements for each quarter of the financial year.^26^ Data on the extended hours access scheme were available for 99.7% (7981/8005) of practices in the General Practice Patient Survey data.

We considered practices that received a payment under the scheme to be scheme participants. All other practices were classed as non-participants, thus creating a binary variable. We could not measure the number of extended opening hours provided over the minimum requirement (30 min per 1000 registered patients) as payments are based solely on the number of patients registered to each practice (multiplied by £1.90).

Participating practices provide appointments outside of the core hours given in their main contracts. Standard core hours are from 08:00 to 18:30, Monday to Friday, for the 95% of practices with General or Personal Medical Services contracts.^27–29^ We excluded the remaining practices as they often open for longer as part of their main contracts,^30^ so not participating in the scheme does not indicate shorter opening hours for them.

### Patient and practice characteristics

We analysed 12 variables as potential confounders. These variables were the main predictors of patient experience in the precursor of the General Practice Patient Survey.^31^

Patient characteristics were age (eight ordinal categories); gender; ethnicity (white, mixed, Asian, black, other^32^); ability to take time off work to see a GP (no, yes, not working); and confidence in managing health (four ordinal categories), as reported in the survey. Socio-economic status was measured in fifths of the national Index of Multiple Deprivation rank for the small areas in which patients lived (lower layer super output areas; mean population of 1500).^33^

Practice characteristics were numbers of registered patients and full-time-equivalent GPs;^34^ national Index of Multiple Deprivation rank for the registered population;^35^ urban/rural location (defined as urban if area population exceeded 10 000^36^); and region of England (of 10 strategic health regions). Clinical quality was assessed using 13 intermediate outcome measures from the UK Quality and Outcomes Framework 2013–2014;^37^ these measures have the largest associations with patient experience of all framework indicators.^38^ We calculated the sum of achievement on the 13 measures, weighted by the relative number of points available.^38^ ^39^

### Statistical methods

All statistical analysis was conducted using Stata MP V.13. We report descriptive statistics for all respondents both unweighted and weighted for survey design and non-response.^21^ We omitted 114 general practices (6809 respondents) that opened or closed within the year, had <1000 registered patients or <50 survey responses (to omit practices with atypical populations).^40^ The number of responses and practices eligible for inclusion for the methods outlined below were 854 206 responses (95% of original sample) and 7428 practices (93% of original sample). When estimating associations, we excluded respondents with missing data for any of the variables in the model (12–15% across experience measures); previous analysis of the General Practice Patient Survey found no difference in results between complete case and multiple imputation approaches.^41^

We estimated the association of participation in the extended hours access scheme with the patient experience measures using three approaches: multilevel random-effects regression, propensity score matching and instrumental variable analysis. These methods are robust under different assumptions, as explained in detail elsewhere.^42^ In each approach, estimates were adjusted for the 12 patient and practice characteristics given above. We adjusted SEs for heteroscedasticity and clustering within practices.

#### Random-effects regression

We first estimated multilevel linear regression models with a random intercept at the general practice level. We tested whether each patient experience measure was significantly different in practices that participated in the scheme versus practices that did not. We also tested whether any differences varied by respondent ability to take time off work to see a GP and by region of England using interactions. Policy to extend opening hours has focused on working people and national implementation.^6^

The random-effects regression models assume that all characteristics of patients and practices that are associated with both scheme participation and patient experience were observed and adjusted for in the models. If this assumption is false, the estimated coefficients will be biased by residual confounding. Some other biases potentially affecting the regression estimates, from misspecification of the form of the outcome equation for example, can be resolved by matching methods.^42^ We therefore used propensity score matching to assess the sensitivity of the results.

#### Propensity score matching

We used logistic regression to estimate the probability that a respondent's general practice participated in the scheme (the propensity score) based on the 12 patient and practice characteristics given above. Each respondent whose practice did participate was then compared with 100 other respondents with the most similar propensity scores whose practices did not participate (nearest neighbours matching with replacement);^43^ matching with 100 respondents rather than the one most similar respondent improved the balance of variables between the participant and non-participant groups. We excluded respondents with no suitable match (due to non-overlapping propensity scores). We used standardised mean differences in patient and practice characteristics between participation groups, before and after matching, to assess matching quality.

The above approaches assume that all variables associated with scheme participation and patient experience are observed and can therefore be accounted for in the models. However, it is plausible that participating practices provide other additional services that we do not have data on but that also affect patient experience, causing estimates to be biased. Instrumental variable analysis can resolve this issue if a valid instrument can be found.

#### Instrumental variable analysis

Valid instruments would influence scheme participation, have no effect on patient experience except through its influence on scheme participation and be unrelated to unobserved confounders.^44^ We considered fifths of the percentage of practices participating in the scheme in each Clinical Commissioning Group (CCG) to be a valid instrument. Such ‘preference-based’ geographic instrumental variables are commonly used.^44^ ^45^

CCGs are groups of general practices (38 on average across 211 CCGs) that plan and commission local health services in England. The percentage of practices that participate in the extended hours access scheme varies considerably across CCGs (10th centile=45%, 90th centile=96%), reflecting different ‘preferences’ of CCGs as largely autonomous organisations. CCGs have a legitimate role in directly influencing member practices on issues such as access, while GPs often feel that their views are not reflected in CCG decisions.^46^ It is plausible that CCGs with similar local populations and practices can have very different views on the extended hours access scheme. This may be due to large variation in governance arrangements of CCGs, their levels of engagement with member practices and who ‘owns’ decision-making.^46^ We examined the association between scheme participation rates within CCGs and practice characteristics to help evaluate instrument validity.

We used two-stage least squares to estimate the effect of the scheme. We report partial R^2^ and F statistics from the first-stage regressions to assess instrument strength. Assuming that the instrumental variable does not modify the effect of scheme participation, the average effect of the scheme on scheme participants is estimated (as for the other two approaches).

## Results

Table 3 describes the 903 357 respondents to the General Practice Patient Survey 2013–2014. Most people were in paid work (57.1% of weighted responses) with a minority unable to take time off work to see a GP (18.7%). Table 4 shows that most respondents were very satisfied (37.2%) or fairly satisfied (42.7%) with the opening hours of their general practices. Results were similar for experience of making an appointment and the overall experience. Table 5 indicates that working people, particularly if they were unable to take time off work to see a GP, reported worse experiences across measures. Mean values of satisfaction with opening hours, experience of making an appointment and overall experience at the practice level were 78.5 (SD 6.3), 76.8 (9.4) and 83.1 (6.5), respectively.

**Table 3:** Characteristics of respondents to the General Practice Patient Survey 2013–2014

Characteristic	Number (unweighted; weighted percentages) of respondents
Age (years)	
18–24	34 815 (3.9; 9.7)
25–34	80 767 (9.1; 17.1)
35–44	111 298 (12.5; 17.3)
45–54	153 641 (17.3; 18.6)
55–64	177 966 (20.0; 14.8)
65–74	183 908 (20.7; 12.3)
75–84	111 332 (12.5; 7.3)
≥85	35 492 (4.0; 2.9)
Total	889 219
Gender	
Male	385 485 (43.3; 49.0)
Female	503 834 (56.7; 51.0)
Total	889 319
Ethnicity	
White	777 904 (87.8; 87.1)
Mixed	6 729 (0.8; 1.0)
Asian	51 629 (5.8; 6.3)
Black	23 581 (2.7; 2.6)
Other	26 215 (3.0; 3.1)
Total	886 058
Socio-economic status*	
1 (most deprived)	186 046 (20.6; 20.6)
2	179 379 (19.9; 20.0)
3	185 234 (20.5; 20.0)
4	181 712 (20.1; 19.7)
5 (least deprived)	170 498 (18.9; 19.8)
Total	902 869
Can take time off work to see general practitioner	
Not working†	460 614 (54.0; 42.9)
Yes	269 493 (31.6; 38.4)
No	122 589 (14.4; 18.7)
Total	852 696
Confident in managing health	
Very	365 679 (42.1; 42.8)
Fairly	436 179 (50.2; 49.7)
Not very	54 953 (6.3; 6.2)
Not at all	11 818 (1.4; 1.3)
Total	868 629

903 357 survey respondents from 8005 general practices; data presented where available for each variable.

Weighted percentages account for survey design and non-response.

*Fifths of the national Index of Multiple Deprivation rank for lower layer super output areas of residence.

†Full-time education, unemployed, sick or disabled, retired, looking after home, other.

**Table 4:** Satisfaction with opening hours, experience of making an appointment and overall experience in the General Practice Patient Survey 2013–2014

Question	Number (unweighted; weighted percentages) of respondents
How satisfied are you with the hours that your general practitioner surgery is open?*	
Very dissatisfied	21 305 (2.5; 3.1)
Fairly dissatisfied	48 015 (5.6; 6.8)
Neither satisfied nor dissatisfied	77 306 (9.0; 10.2)
Fairly satisfied	352 262 (41.1; 42.7)
Very satisfied	358 987 (41.8; 37.2)
Total	857 875
Overall, how would you describe your experience of making an appointment?	
Very poor	26 881 (3.1; 4.1)
Fairly poor	50 875 (5.9; 7.4)
Neither good nor poor	99 458 (11.6; 13.9)
Fairly good	334 833 (39.0; 40.9)
Very good	346 279 (40.3; 33.8)
Total	858 326
Overall, how would you describe your experience of your general practitioner surgery?	
Very poor	8 146 (0.9; 1.2)
Fairly poor	25 043 (2.8; 3.6)
Neither good nor poor	69 618 (7.9; 9.5)
Fairly good	342 015 (38.7; 42.6)
Very good	437 868 (49.6; 43.1)
Total	882 690

903 357 survey respondents from 8005 general practices; data presented where available for each variable.

Weighted percentages account for survey design and non-response.

*Responses of ‘I'm not sure when my GP surgery is open’ were excluded (n=25 271).

**Table 5:** Mean satisfaction with opening hours, experience of making an appointment and overall experience in the General Practice Patient Survey 2013–2014, by ability to take time off work to see a general practitioner (GP)

	Satisfaction with opening hours	Experience of making an appointment	Overall experience
Overall	78.4	76.8	83.2
Can take time off work to see a GP:			
Not working*	82.6	80.3	86.2
Yes	77.1	76.3	82.4
No	65.3	65.0	74.2

Mean values of outcome measures are on a 0–100 scale.

Table based on 852 696 survey responses with non-missing data for row variables; data presented where available for outcome measures (n≥811 589 responses).

*Full-time education, unemployed, sick or disabled, retired, looking after home, other.

**Figure 1:**
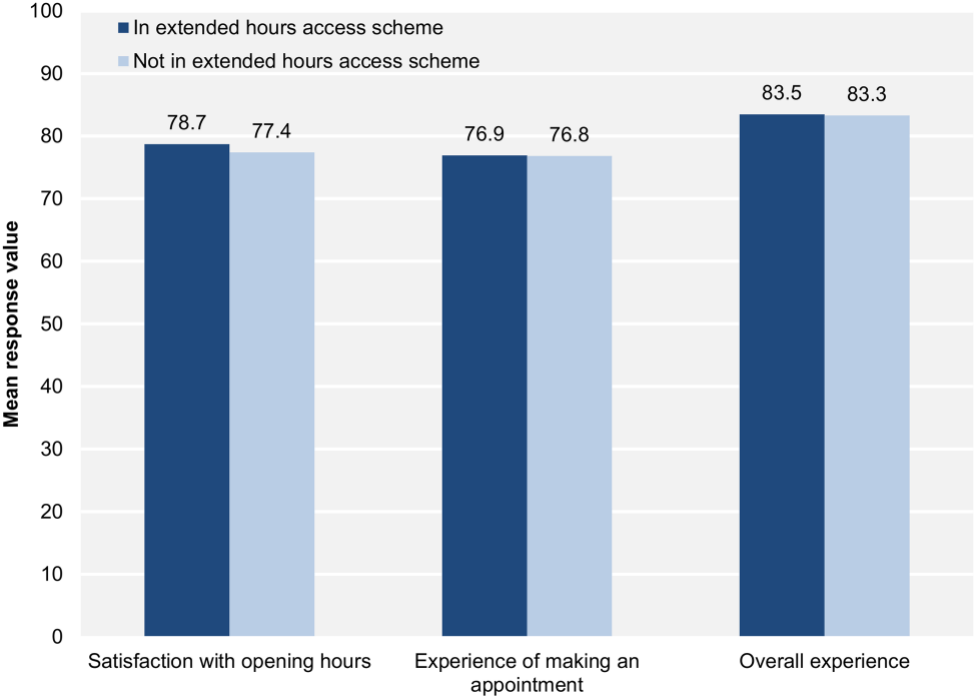
Mean satisfaction with opening hours, experience of making an appointment and overall experience by scheme participation.

Most of the included general practices participated in the extended hours access scheme in 2013–2014 (73.9%; 5492/7428). The mean payment to participating practices was £10 454 (IQR £5863–16 772). Participation rates and other characteristics of General Medical Services practices were comparable to those of Personal Medical Services practices (see online supplementary appendix 1). Figure 1 shows how mean values of the patient experience measures did not differ much by scheme participation. Table 6 presents estimates of adjusted mean differences, from random-effects regression, propensity score matching and instrumental variable analysis.

10.1136/bmjqs-2016-005233.supp1 Supplementary appendices

**Table 6:** Adjusted associations between participation in the extended hours access scheme and patient experience, using multilevel random-effects regression models, propensity score matching and instrumental variable analysis

	Satisfaction with opening hours	Experience of making an appointment	Overall experience
Number of responses	731 700	725 885	753 020
Number of general practices	7399	7399	7399
Between-practice SD*	4.5	7.0	4.3
Random-effects regression models			
Mean difference (95% CI)	1.25 (0.96 to 1.55)	0.48 (0.07 to 0.90)	0.32 (0.04 to 0.60)
p Value	<0.001	0.022	0.026
Standardised mean difference	0.28	0.07	0.07
Propensity score matching			
Mean difference (95% CI)	1.35 (1.00 to 1.70)	0.51 (−0.03 to 1.04)	0.39 (0.03 to 0.74)
p Value	<0.001	0.063	0.032
Standardised mean difference	0.30	0.07	0.09
Instrumental variable analysis			
Mean difference (95% CI)	1.36 (0.71 to 2.00)	1.79 (0.84 to 2.75)	1.13 (0.50 to 1.76)
p Value	<0.001	<0.001	<0.001
Standardised mean difference	0.30	0.25	0.26

Numbers of responses and general practices correspond to respondents with no missing data for relevant outcome models.

Mean differences are relative to the means for general practices not participating in the scheme.

All models adjusted for/balanced: respondent age, gender, ethnicity, socio-economic status, ability to take time off work to see a GP, confidence in managing health; and general practice registered population size, number of full-time-equivalent GPs per 10 000 patients, socio-economic status of registered population, Quality and Outcomes Framework achievement, urban/rural location and region of England.

*SD of practice-level random effects adjusted for patient characteristics only. Standardised mean differences equal mean differences divided by this SD.

### Random-effects regression

In the multilevel random-effects regression models, respondents registered to general practices participating in the scheme reported greater satisfaction with opening hours on average (mean difference 1.25, 95% CI 0.96 to 1.55). The standardised mean difference of 0.28 indicates a small association with satisfaction. The scheme was estimated to have minimal associations with experience of making an appointment (0.48, 0.07 to 0.90) and overall experience (0.32, 0.04 to 0.60); the mean differences correspond to 0.07 SDs in these measures of patient experience at the practice level.

**Figure 2:**
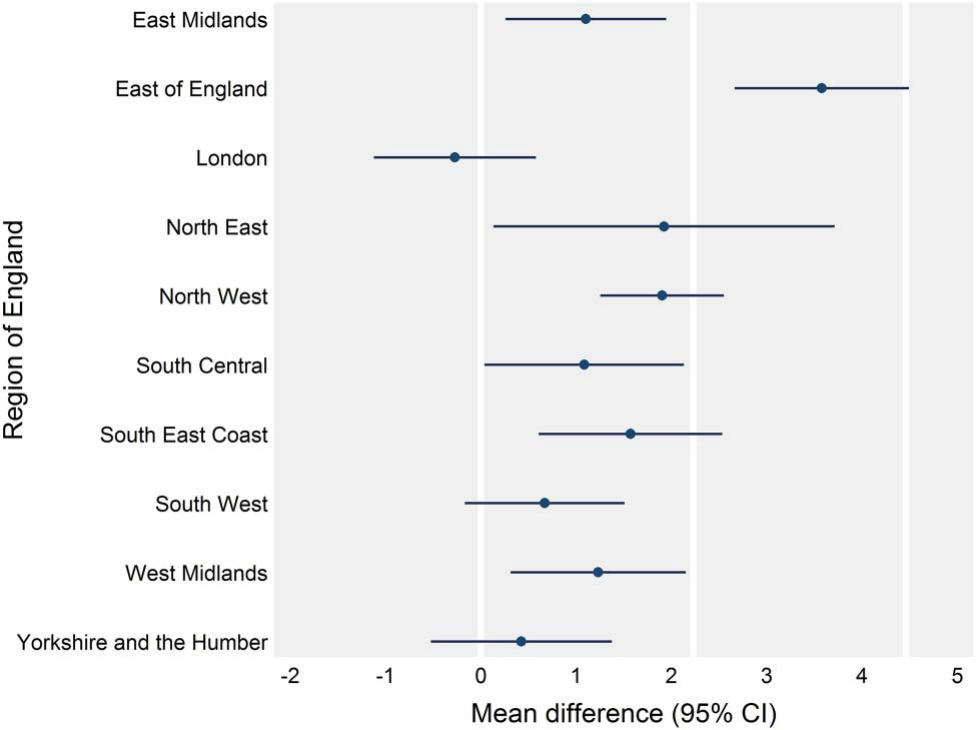
Associations of the extended hours access scheme with satisfaction with opening hours by region of England estimated using multilevel random effects regression models. Plotted estimates are adjusted mean differences and bars represent 95% CIs. Reference lines are at 0, 0.5 (2.23) and 1 (4.46) SDs in satisfaction with opening hours at the practice level (see table 6).

The association of scheme participation with satisfaction with opening hours differed according to respondent ability to take time off work to see a GP (p<0.001 for joint test of interactions). Table 7 shows a greater association for those unable to take time off (standardised mean difference 0.47) compared with respondents who were not in paid work (0.20). There was no evidence of this effect modification for experience of making an appointment or overall experience (p=0.315 and 0.788). Figure 2 presents how the association for satisfaction with opening hours varied by region of England (p<0.001). The largest association was seen for the East of England (mean difference 3.58, 2.66 to 4.49), while there was no evidence of an association in London (−0.27, −1.12 to 0.58; p=0.531). Mean differences typically remained minimal across regions for the other two experience measures (see online supplementary appendix 2).

**Table 7:** Associations of the extended hours access scheme with patient experience by ability to take time off work to see a general practitioner (GP), estimated using multilevel random-effects regression models

	Satisfaction with opening hours	Experience of making an appointment	Overall experience
Cannot take time off			
Mean difference (95% CI)	2.08 (1.53 to 2.63)	0.65 (0.04 to 1.26)	0.27 (−0.18 to 0.71)
p Value	<0.001	0.035	0.237
Standardised mean difference	0.47	0.09	0.06
Can take time off			
Mean difference (95% CI)	1.52 (1.16 to 1.89)	0.57 (0.10 to 1.03)	0.37 (0.04 to 0.69)
p Value	<0.001	0.017	0.028
Standardised mean difference	0.34	0.08	0.09
Not working*			
Mean difference (95% CI)	0.87 (0.60 to 1.15)	0.39 (−0.01 to 0.79)	0.30 (0.03 to 0.57)
p Value	<0.001	0.057	0.028
Standardised mean difference	0.20	0.06	0.07

Categories based on responses to ‘If you need to see a GP at your GP surgery during your typical working hours, can you take time away from your work to do this?’.

Model specification is the same as for random effects regression model in table 6 with interaction terms added between participation in the extended hours access scheme and ability to take time off work to see a GP.

p Values for joint tests of interaction terms were <0.001 (opening hours), 0.315 (appointment) and 0.788 (overall).

*Full-time education, unemployed, sick or disabled, retired, looking after home, other.

Respondents who were unable to take time off work to see a GP or were not at all confident in managing their health reported substantially worse patient experiences (mean differences relative to those not working or those very confident in managing their health ≤−7.96; see online supplementary appendix 3). For example, mean satisfaction with opening hours for respondents who could not take time off work to see a GP was 14.62 (14.40 to 14.83) lower than for respondents who were not working. Patients who could take time off to see a GP responded more similarly to those not working across measures (eg, satisfaction with opening hours −3.68, −3.82 to −3.53).

### Propensity score matching

General practices were more likely to participate in the extended hours access scheme if they had larger registered populations (OR for an SD increase 1.26, 1.18 to 1.35) or performed better in the Quality and Outcomes Framework (1.22, 1.15 to 1.28; see online supplementary appendix 4). Scheme participation was highest in the northeast of England (relative to East Midlands, 5.51, 3.85 to 7.90). Before matching, the largest standardised mean difference in a variable between the two participation groups was 18.5% (percentage of respondents in the north east of England); most variables were highly balanced (average standardised mean difference 4.5%).

Using propensity score matching, participation in the extended hours access scheme was associated with a 1.35 (1.00 to 1.70) increase in satisfaction with opening hours (table 6). This estimate and those for the two other experience measures were similar to those from the random-effects regression models, thus supporting the specification of these regression models. Propensity score matching, in general, reduced the standardised mean differences in characteristics of the two participation groups; the average difference reduced to 0.5%, indicating good matching quality (see online supplementary appendix 5). A suitable match could not be found for a very small number of respondents (0.09%).

### Instrumental variable analysis

The percentage of practices that participated in the scheme varied from 34.9% to 96.0% across categories of the instrumental variable (fifths of the percentage of practices participating in the scheme in each CCG). This variable explained 18.5% of variation in the probability that a respondent was registered to a participating practice (partial R^2^=0.185; F statistic ≥1702). Other characteristics of general practices were generally similar across categories of the instrumental variable (see online supplementary appendix 6).

In the instrumental variable analysis, the estimated effect of scheme participation on mean satisfaction with opening hours was 1.36 (0.71 to 2.00) with a corresponding standardised mean difference of 0.30, indicating a small effect on satisfaction (table 6). The mean differences for experience of making an appointment (1.79, 0.84 to 2.75) and overall experience (1.13, 0.50 to 1.76) were larger than as estimated in the regression models and using propensity score matching, but effect sizes remained small (standardised mean differences 0.25 and 0.26).

## Discussion

In the General Practice Patient Survey 2013–2014, most respondents were satisfied with the opening hours of their general practices and had good experiences of making an appointment and of their practices overall. Most general practices participated in the extended hours access scheme. Random-effects regression models, propensity score matching and instrumental variable analysis all estimated the associations between scheme participation and patient experience measures to be positive but small. The association with satisfaction with opening hours was greatest for employed respondents who were unable to take time off work to see a GP, but this group still had substantially worse experiences across all measures. Results were generally consistent across regions of England.

### Strengths and limitations

We suggest four strengths of the study. First, the study addresses two of the most prominent topics in current health policy in England—extended opening hours and patient experience of general practice. Second, we evaluated their relation using national data sets that include almost all general practices, such that the results are highly relevant to central government policy. The General Practice Patient Survey itself is monitored by government to assess NHS performance^20^ and by practices to set opening hours under the extended hours access scheme.

Third, extended opening hours policies remain largely unevaluated. This paper is the first national analysis of such a policy and the first to use general practice payment data in this context. Fourth, using the multilevel structure of available data, we adjusted results for observed differences between patients and general practices and also tried to adjust for unobserved differences in the instrumental variable analysis. We thus tested the results' sensitivity to model assumptions and found consistent results across models.

A limitation was that the payment data do not indicate exactly when practices were extending their opening hours during the week. To our knowledge, valid national data on the exact opening times of practices do not exist; data reported by practices on an NHS information website are inaccurate.^9^ A telephone survey of a nationally representative 4% sample of practices suggests that around half of extended hours with GP face-to-face consultations are after 18:30 on weekdays (1.4 of 2.6 hours each week; 0.9 hours at weekends; 0.3 hours before 08:00).^9^ Other health professionals are also eligible to provide consultations under the scheme.

Cross-sectional studies are often limited by residual confounding. In this study, observed characteristics of patients and general practices were very similar between scheme participants and non-participants. Given this fact and the high rate of scheme participation, we do not expect potential sources of residual confounding to have important effects on the results. This includes possible non-response bias in the General Practice Patient Survey; response rates were similar between participation groups (36.9% and 37.2%). Instrumental variable analysis, which attempts to explicitly address unmeasured confounding, produced similar results to other approaches.

Several explanations for the results that do not relate solely to the effect of scheme participation cannot be ruled out using a cross-sectional design. The scheme's introduction in 2008 could have improved patient experience in the short term, with extended opening hours increasingly part of normal expectations over time such that the effect has since reduced. Experiences in participating practices before they joined the scheme may also have been worse than the current experiences in non-participating practices. These alternative explanations, if true, would mean that the results presented underestimate the effect of the extended hours access scheme. A longitudinal study design was not feasible given the novelty of the extended hours access scheme data.

The analysis was limited to three experience measures that we thought were the most relevant to current policy. Associations with other experience measures may differ, however. In a supplementary analysis (requested in the journal review process), we estimated a multilevel regression model for an additional outcome measure relating to appointment convenience. This measure had five levels: no appointment, not at all convenient, not very convenient, fairly convenient and very convenient (interval scale from 0 to 100). The adjusted association with scheme participation was 0.55 (95% CI 0.21 to 0.90), again indicating a minimal difference.

### Relation to existing literature

Most respondents to the General Practice Patient Survey 2013–2014 found current opening times convenient (79.9% of weighted sample).^47^ The percentage reporting both that they were inconvenient and additional opening times on Saturdays would be helpful was 14.9%, while it was 7.4% for Sundays;^47^ this does not necessarily mean that only a minority of patients would benefit from extending opening hours, however. Patients who cannot take time off work to see a GP are particularly less likely to find current opening times convenient,^48^ yet most of these respondents still do so (55.8%).^49^ Those who can take time off work to see a GP are also less likely to find current times convenient (77.7%) than people not in paid work (91.4%).^49^ Many measures of patient experience in the General Practice Patient Survey have worsened year-on-year since 2011–2012.^47^ ^50^ In international patient surveys, the timeliness of primary care in England still ranks highly.^51^

Patients who cannot take time off work to see a GP have long reported worse experiences of their general practices in national surveys. In 2007–2008, these patients reported being less able to get an appointment and to see a particular GP, as well as worse satisfaction with opening hours.^31^ Our results suggest that the extended hours access scheme might reduce some of these differentials but is unlikely to resolve them entirely. Respondents registered to practices with larger populations or located in certain regions, particularly London, also gave substantially more negative responses in 2007–2008.^31^ These findings are again consistent with our results. Other patient characteristics, such as younger age, also demonstrate consistent negative associations across experience measures.^23^ ^31^ ^41^

Overall experience was more strongly associated with the quality of doctor communication than the timing of appointments (within or more than two weekdays ahead) in the General Practice Patient Survey 2009–2010.^52^ Discrete choice experiments suggest that patients often give less weight to timings of appointments than other characteristics such as seeing a particular doctor.^53–56^ This may help explain why participation in the extended hours access scheme had a limited association with overall experience in our study. A previous analysis aimed to determine the effect of the introduction of the extended hours access scheme in 2008 in 13 of 152 areas of England, but its validity is compromised by the unreliable data sources used (such as internet searches).^57^

The UK health secretary has stated that ‘the role and purpose of seven day primary care is about much more than convenience—it is about making sure precious hospital capacity is kept clear for those who really need it’.^2^ Several national studies of the General Practice Patient Survey linked to hospital data suggest that general practices with greater access (relating here to the ability to get an appointment) have lower adjusted rates of both emergency department visits and emergency hospital admissions.^58–68^ However, the extent to which residual confounding explains these results is unknown; more robust longitudinal analyses are needed.^47^ There is likely to be much variability in the extent extended opening hours schemes improve access and for whom.

Programmes to extend opening hours in Manchester had a limited effect on use of emergency departments and patient experience, with results changing across model specifications and by local area.^69^ In London, four general practices that began opening 7 days a week reduced use of emergency departments relative to a local control group.^70^ The national evaluation of the first wave of the prime minister's GP access fund did not use methods that were adequate to determine its true effect on patient experience or use of hospital services.^71^ It did, however, identify little demand for appointments on Sundays in most pilots with some no longer opening on these days.^71^ National research funders should commission relevant academic evaluations.

### Policy implications

Our results suggest that the extended hours access scheme has a limited association with patient experience. Assuming that this association represents the true effect of the scheme, possible explanations include that it is difficult to improve experiences beyond existing levels (diminishing marginal returns) and that some participating practices are redistributing appointments rather than offering more of them (contrary to scheme requirements). Alternatively, patients who use extra appointments may often not reflect this in their reported experiences despite the benefits gained; critical respondents, for example, may remain negative for reasons besides service provision. Many patients may simply be unaware of their practices' opening times, preventing extended hours from translating into improved satisfaction. The extra appointments may also be used by patients whom the intervention is not targeted at, such as those not in full-time work, who could otherwise get an appointment for another time. Our results do suggest that patients who are unable to take time off work to see a GP could benefit more from extended opening hours, however, which supports the mechanism expected to link extended hours to improved satisfaction in the General Practice Patient Survey.

The modest associations reported could instead be due to the limited size of the intervention—the minimum requirement of 30 min of additional appointments per 1000 registered patients each week is not a large change to opening hours for an average practice (equivalent to 3 hours and 45 min). It may represent an even smaller change to the number of additional consultations provided, and these consultations may not be with patients' preferred health professionals. Since additional appointments can be provided concurrently (for at least 30 min) to meet the minimum requirement, actual opening times may also not change that much. Revisions to the scheme may improve its benefit.

The results are also relevant to the prime minister's GP access fund (table 1a). In the first 20 pilot areas of the GP access fund, medium-sized pilots provided around 41 min of extended hours per 1000 registered patients each week.^71^ This is comparable to the minimum amount required by the extended hours access scheme alone, such that the effect on patient experience may be similarly limited. Moreover, the additional appointments can be provided collectively between practices working in groups in the pilot areas, in contrast to each practice individually extending opening hours under the extended hours access scheme 2013–2014. Therefore, patients may have to attend practices other than their own in pilot areas. These facts question the expected impact of extended opening hours on patient experience in the GP access fund pilots. Other interventions trialled in these pilots may contribute to any effects though, and other rationales besides improving patient experience exist.^71^

The national evaluation of the GP access fund pilot areas reported that 75% of appointments outside core times were used. It therefore cautiously suggested that around 30 min of extended hours per 1000 population per week would be optimal.^71^ This is the minimum set in the extended hours access scheme, which suggests that this scheme may be sufficient alone to extend hours suitably. It remains to be seen whether utilisation increases with time, however, and what the results from the second wave of pilot areas are.

Government plans for access to general practice appear unlikely to change soon. When once asked about the aim of 7-day working, the UK health secretary replied, ‘Increasing convenience for the general public in terms of being able to make routine evening and weekend appointments is a manifesto commitment that this government made, so we have to honour that’.^1^ Improving patient experience has been given as one of three key objectives for these changes.^71^ In conclusion, this study questions whether large improvements in patient experience will be achieved through existing changes to opening hours alone.
