# Supplementary material for: Extended opening hours and patient experience of general practice in England: multilevel regression analysis of a national patient survey
Source: BMJ Qual Saf. 2016 Jun 24;26(5):360–71. doi: 10.1136/bmjqs-2016-005233 (PMC5530331; doi:10.1136/bmjqs-2016-005233)
Supplement: Supplementary appendices [file bmjqs-2016-005233supp001.pdf]

Appendices

Appendix 1 | Characteristics of included general practices overall and by contract type

	Overall (n=7428)	General Medical Services (n=4283)	Personal Medical Services (n=3145)
Extended hours access scheme:			
Participation rate	73.9	74.3	73.5
Payment of participants (£)	10 454 (5863 to 16 772)	10 279 (5945 to 16 024)	10 696 (5766 to 17 974)
Registered population size	6416 (3867 to 9727)	6149 (3680 to 9171)	6871 (4153 to 10 411)
Number of GP FTEs per 10 000 registered patients	5.9 (4.8 to 7.3)	5.8 (4.7 to 7.2)	6.1 (4.9 to 7.6)
National Index of Multiple Deprivation rank*	4161 (2186 to 6094)	4411 (2377 to 6287)	3874 (1929 to 5790)
Quality and Outcomes Framework achievement†	75.9 (72.3 to 79.1)	76.1 (72.5 to 79.4)	75.6 (72.0 to 78.7)
Urban location (vs. rural)	83.8	81.6	86.8
Region of England:			
East Midlands	7.9	7.3	8.7
East of England	9.8	9.9	9.6
London	16.8	14.4	20.1
North East	5.0	3.4	7.1
North West	15.3	17.7	12.1
South Central	6.4	8.1	4.1
South East Coast	7.9	10.5	4.3
South West	9.2	7.2	12.0
West Midlands	11.9	13.2	10.1
Yorkshire and the Humber	9.9	8.3	12.1

GP FTE=general practitioner full-time equivalent.
Statistics are medians (interquartile range) for continuous variables and percentages for categorical variables.
*Ranges from 1 (most deprived) to 7997 (least deprived) where each general practice has a different rank.
†Sum of achievement (proportion of patients for whom the outcome was met) on 13 intermediate outcome measures, weighted by the relative number of points available for each measure in the Quality and Outcomes Framework 2013-14.

Appendix 2 | Associations of the extended hours access scheme with patient experience by region of England, estimated using multilevel random effects regression models. Figures are adjusted mean differences (95% confidence intervals)

Region	Satisfaction with opening hours	Experience of making an appointment	Overall experience
East Midlands	1.10 (0.26 to 1.95)	-0.29 (-1.58 to 1.00)	0.16 (-0.68 to 1.01)
East of England	3.58 (2.66 to 4.49)	1.97 (0.64 to 3.30)	1.27 (0.37 to 2.16)
London	-0.27 (-1.12 to 0.58)	-0.59 (-1.65 to 0.47)	-0.49 (-1.25 to 0.28)
North East	1.92 (0.13 to 3.71)	1.34 (-0.98 to 3.65)	1.11 (-0.47 to 2.68)
North West	1.90 (1.25 to 2.55)	0.87 (-0.15 to 1.88)	0.33 (-0.32 to 0.97)
South Central	1.08 (0.04 to 2.13)	2.21 (0.46 to 3.96)	1.31 (0.22 to 2.41)
South East Coast	1.57 (0.61 to 2.53)	0.87 (-0.49 to 2.23)	0.42 (-0.55 to 1.40)
South West	0.67 (-0.17 to 1.51)	0.21 (-1.03 to 1.46)	0.31 (-0.57 to 1.20)
West Midlands	1.23 (0.31 to 2.15)	0.07 (-1.06 to 1.20)	0.13 (-0.67 to 0.93)
Yorkshire and the Humber	0.43 (-0.52 to 1.37)	-0.20 (-1.57 to 1.16)	0.02 (-0.82 to 0.86)

Model specification is the same as in table 6 with interaction terms added between participation in the extended hours access scheme and region of England.

P values for joint tests of interaction terms were <0.001 (opening hours), 0.049 (appointment), and 0.139 (overall).

Appendix 3 | Associations of patient experience with characteristics of patients and general practices, estimated using multilevel random effects regression models. Figures are adjusted mean differences (95% confidence intervals)

	Satisfaction with opening hours	Experience of making an appointment	Overall experience
Age (years):			
18 to 24	(ref)	(ref)	(ref)
25 to 34	2.65 (2.29 to 3.02)	2.87 (2.52 to 3.23)	2.12 (1.81 to 2.42)
35 to 44	4.28 (3.94 to 4.63)	4.81 (4.46 to 5.15)	5.16 (4.87 to 5.44)
45 to 54	4.86 (4.52 to 5.20)	5.36 (5.03 to 5.69)	7.11 (6.83 to 7.38)
55 to 64	6.18 (5.85 to 6.52)	6.87 (6.55 to 7.20)	8.45 (8.18 to 8.72)
65 to 74	8.46 (8.13 to 8.80)	9.57 (9.24 to 9.90)	10.66 (10.39 to 10.94)
75 to 84	10.30 (9.95 to 10.64)	12.51 (12.17 to 12.85)	13.32 (13.03 to 13.60)
85 or over	10.12 (9.72 to 10.52)	14.04 (13.64 to 14.45)	14.14 (13.81 to 14.47)
Gender:			
Male	(ref)	(ref)	(ref)
Female	1.15 (1.05 to 1.26)	-0.61 (-0.72 to -0.50)	-0.15 (-0.24 to -0.07)
Ethnicity:			
White	(ref)	(ref)	(ref)
Mixed	0.20 (-0.47 to 0.87)	0.06 (-0.63 to 0.75)	0.19 (-0.37 to 0.75)
Asian	-1.59 (-1.89 to -1.29)	-3.69 (-4.01 to -3.36)	-2.31 (-2.56 to -2.05)
Black	3.02 (2.64 to 3.41)	2.51 (2.11 to 2.91)	2.65 (2.32 to 2.98)
Other	3.14 (2.77 to 3.50)	1.25 (0.86 to 1.65)	1.34 (1.03 to 1.66)
Socioeconomic status:*			
1 (most deprived)	(ref)	(ref)	(ref)
2	-1.32 (-1.51 to -1.13)	-0.55 (-0.75 to -0.35)	-0.58 (-0.74 to -0.42)
3	-2.27 (-2.47 to -2.07)	-0.81 (-1.02 to -0.59)	-1.05 (-1.22 to -0.88)
4	-2.93 (-3.14 to -2.71)	-0.94 (-1.17 to -0.72)	-1.36 (-1.54 to -1.19)
5 (least deprived)	-3.65 (-3.89 to -3.42)	-1.26 (-1.50 to -1.02)	-1.70 (-1.89 to -1.51)
Can take time off work to see GP:			
Not working	(ref)	(ref)	(ref)
Yes	-3.68 (-3.82 to -3.53)	-1.89 (-2.04 to -1.74)	-1.56 (-1.68 to -1.44)
No	-14.62 (-14.83 to -14.40)	-11.04 (-11.25 to -10.83)	-7.96 (-8.13 to -7.80)
Confident in managing health:			
Very	(ref)	(ref)	(ref)
Fairly	-7.26 (-7.37 to -7.15)	-7.58 (-7.70 to -7.47)	-7.23 (-7.32 to -7.14)
Not very	-11.55 (-11.81 to -11.29)	-13.84 (-14.11 to -13.56)	-13.18 (-13.41 to -12.94)
Not at all	-10.14 (-10.73 to -9.55)	-12.25 (-12.86 to -11.63)	-12.64 (-13.19 to -12.09)
In extended hours access scheme	1.25 (0.96 to 1.55)	0.48 (0.07 to 0.90)	0.32 (0.04 to 0.60)
Registered population size†	-0.79 (-0.93 to -0.66)	-3.31 (-3.55 to -3.07)	-1.12 (-1.26 to -0.99)
Number of GP FTEs per 10 000 patients†	0.61 (0.45 to 0.77)	0.90 (0.70 to 1.09)	0.81 (0.66 to 0.95)
National Index of Multiple Deprivation rank of practice†	0.02 (-0.14 to 0.17)	1.29 (1.07 to 1.52)	1.27 (1.12 to 1.42)
Quality and Outcomes Framework achievement†	0.49 (0.36 to 0.63)	0.97 (0.77 to 1.16)	0.65 (0.52 to 0.78)
Practice location:			
Urban	(ref)	(ref)	(ref)
Rural	-0.50 (-0.87 to -0.14)	1.85 (1.35 to 2.35)	0.73 (0.38 to 1.07)
Region of England:			
East Midlands	(ref)	(ref)	(ref)
East of England	-0.95 (-1.52 to -0.38)	1.29 (0.44 to 2.14)	0.06 (-0.51 to 0.63)
London	-3.12 (-3.65 to -2.58)	-1.32 (-2.11 to -0.54)	-1.82 (-2.36 to -1.29)
North East	1.37 (0.73 to 2.02)	2.43 (1.45 to 3.42)	2.08 (1.44 to 2.72)
North West	0.30 (-0.21 to 0.82)	0.94 (0.15 to 1.73)	1.55 (1.04 to 2.06)
South Central	-0.91 (-1.51 to -0.30)	1.16 (0.20 to 2.11)	0.18 (-0.44 to 0.80)
South East Coast	-2.68 (-3.29 to -2.07)	0.10 (-0.80 to 1.00)	-0.41 (-1.03 to 0.21)
South West	0.36 (-0.18 to 0.90)	3.37 (2.56 to 4.18)	2.06 (1.52 to 2.59)
West Midlands	-1.50 (-2.06 to -0.93)	0.36 (-0.45 to 1.17)	0.17 (-0.38 to 0.71)
Yorkshire and the Humber	-0.39 (-0.95 to 0.17)	0.76 (-0.09 to 1.61)	1.09 (0.55 to 1.63)

FTE=full time equivalent; GP=general practitioner.
*Fifths of the national Index of Multiple Deprivation rank for lower layer super output areas of residence.
†Estimates are for a standard deviation increase in the characteristic at the practice level.

Appendix 4 | Associations between participation in the extended hours access scheme and characteristics of patients and general practices, estimated using logistic regression

	Odds ratio	P value	95% confidence interval
Age (years):			
18 to 24	1		
25 to 34	0.99	0.708	0.96 to 1.03
35 to 44	1.01	0.596	0.98 to 1.04
45 to 54	1.02	0.136	0.99 to 1.06
55 to 64	1.05	0.006	1.01 to 1.08
65 to 74	1.06	<0.001	1.03 to 1.10
75 to 84	1.05	0.018	1.01 to 1.09
85 or over	1.05	0.028	1.01 to 1.10
Gender:			
Male	1		
Female	1.00	0.800	0.99 to 1.01
Ethnicity:			
White	1		
Mixed	0.90	0.004	0.84 to 0.97
Asian	1.04	0.450	0.94 to 1.14
Black	0.86	0.003	0.79 to 0.95
Other	0.89	0.003	0.83 to 0.96
Socioeconomic status:*			
1 (most deprived)	1		
2	1.15	<0.001	1.08 to 1.23
3	1.16	<0.001	1.08 to 1.25
4	1.17	<0.001	1.10 to 1.23
5 (least deprived)	1.00	0.951	0.95 to 1.05
Can take time off work to see GP:			
Not working	1		
Yes	0.99	0.097	0.97 to 1.00
No	1.01	0.569	0.98 to 1.03
Confident in managing health:			
Very	1		
Fairly	0.97	<0.001	0.96 to 0.98
Not very	0.95	<0.001	0.93 to 0.98
Not at all	0.97	0.276	0.93 to 1.02
Registered population size†	1.26	<0.001	1.18 to 1.35
Number of GP FTEs per 10 000 patients†	0.99	0.779	0.94 to 1.05
National Index of Multiple Deprivation rank of practice†	0.89	<0.001	0.84 to 0.95
Quality and Outcomes Framework achievement†	1.22	<0.001	1.15 to 1.28
Practice location:			
Urban	1		
Rural	0.79	0.004	0.68 to 0.93
Region of England:			
East Midlands	1		
East of England	2.86	<0.001	2.23 to 3.67
London	3.00	<0.001	2.40 to 3.76
North East	5.51	<0.001	3.85 to 7.90
North West	1.71	<0.001	1.38 to 2.12
South Central	2.31	<0.001	1.75 to 3.04
South East Coast	1.47	0.002	1.15 to 1.87
South West	4.19	<0.001	3.21 to 5.47
West Midlands	2.23	<0.001	1.77 to 2.82
Yorkshire and the Humber	2.42	<0.001	1.89 to 3.08

FTE=full time equivalent; GP=general practitioner.
758 610 observations; standard errors adjusted for clustering in 7399 general practices.
*Fifths of the national Index of Multiple Deprivation rank for lower layer super output areas of residence.
†Estimates are for a standard deviation increase in the characteristic at the practice level.

Appendix 5 | Differences in means of observed characteristics between participation groups before (U) and after (M) propensity score matching

		Unmatched (U)/Matched (M)	In extended hours access scheme	Not in extended hours access scheme	Standardised difference
**Age (years):**	25 to 34	U	0.090	0.089	0.0
		M	0.090	0.089	0.1
	35 to 44	U	0.125	0.126	-0.4
		M	0.125	0.126	-0.2
	45 to 54	U	0.175	0.178	-0.6
		M	0.175	0.175	0.0
	55 to 64	U	0.205	0.204	0.3
		M	0.205	0.205	0.1
	65 to 74	U	0.209	0.207	0.5
		M	0.209	0.209	0.1
	75 to 84	U	0.121	0.121	0.2
		M	0.121	0.121	0.1
	85 or over	U	0.036	0.036	0.2
		M	0.036	0.036	0.0
**Gender:**	Female	U	0.560	0.559	0.3
		M	0.560	0.559	0.2
**Ethnicity:**	Mixed	U	0.007	0.007	-0.2
		M	0.007	0.007	0.0
	Asian	U	0.057	0.051	2.4
		M	0.057	0.058	-0.6
	Black	U	0.023	0.023	0.0
		M	0.023	0.023	-0.3
	Other	U	0.026	0.026	-0.5
		M	0.026	0.026	-0.3
**Socioeconomic status:**	2	U	0.200	0.177	6.0
		M	0.200	0.200	0.1
	3	U	0.212	0.200	3.1
		M	0.212	0.211	0.3
	4	U	0.209	0.207	0.4
		M	0.209	0.209	-0.1
	5 (most deprived)	U	0.190	0.225	-8.5
		M	0.190	0.188	0.5
**Can take time off work to see GP:**	Yes	U	0.319	0.325	-1.2
		M	0.319	0.319	-0.1
	No	U	0.144	0.142	0.6
		M	0.144	0.144	0.1
**Confident in managing health:**	Fairly	U	0.503	0.507	-0.9
		M	0.503	0.503	-0.2
	Not very	U	0.059	0.060	-0.2
		M	0.059	0.060	-0.2
	Not at all	U	0.012	0.012	0.2
		M	0.012	0.012	0.1

**Registered population size***		U	0.087	-0.089	17.7
		M	0.081	0.097	-1.6
**Number of GP FTEs per 10 000 patients***		U	0.016	-0.015	3.0
		M	0.016	-0.002	1.8
**Deprivation rank of registered population***		U	0.052	0.130	-7.8
		M	0.052	0.041	1.1
**Quality and Outcomes Framework achievement***		U	0.039	-0.075	11.4
		M	0.039	0.044	-0.5
**Practice location:**	Rural	U	0.163	0.212	-12.5
		M	0.163	0.168	-1.1
**Region of England:**	East of England	U	0.106	0.092	4.8
		M	0.106	0.108	-0.7
	London	U	0.167	0.125	12.2
		M	0.168	0.170	-0.7
	North East	U	0.059	0.023	18.5
		M	0.059	0.054	2.3
	North West	U	0.141	0.176	-9.7
		M	0.141	0.138	0.8
	South Central	U	0.067	0.065	0.6
		M	0.067	0.070	-1.4
	South East Coast	U	0.071	0.113	-14.5
		M	0.071	0.070	0.4
	South West	U	0.109	0.064	16.1
		M	0.109	0.109	0.1
	West Midlands	U	0.115	0.114	0.2
		M	0.115	0.113	0.5
	Yorkshire and the Humber	U	0.102	0.093	3.1
		M	0.102	0.101	0.3

Mean absolute standardised difference before and after matching was 4.5% and 0.5%, respectively.
*Variables are standardised at the general practice level.

Appendix 6 | Associations between characteristics of general practices and fifths of Clinical Commissioning Group level participation in the extended hours access scheme (the instrumental variable)

Category of instrumental variable	Percentage of practices participating in the scheme	Registered population size	Number of GP FTEs per 10 000 patients	Deprivation rank of registered population	Quality and Outcomes Framework achievement	Percentage of practices in urban areas
1	34.9%	7326	6.1	4433	75.4	79.8%
2	69.7%	7056	6.2	4065	75.7	82.5%
3	78.8%	7014	6.4	3453	75.7	82.4%
4	88.1%	7138	6.3	4404	75.5	84.0%
5	96.0%	7570	6.4	4170	75.2	90.1%

FTE=full time equivalent; GP=general practitioner.
Clinical Commissioning Groups are local groups of general practices that plan and commission hospital services and some community services for their populations. There were 211 of these groups in 2013-14 each consisting of 38 member practices on average.
